# The Complex Clinical Issues Involved in an Athlete’s Decision to Retire from Collision Sport Due to Multiple Concussions: A Case Study of a Professional Athlete

**DOI:** 10.3389/fneur.2013.00141

**Published:** 2013-09-27

**Authors:** Andrew Gardner

**Affiliations:** ^1^Centre for Translational Neuroscience and Mental Health, School of Medicine and Public Health, University of Newcastle, Callaghan, NSW, Australia

**Keywords:** case study, brain concussion, athlete, sport, retirement

## Abstract

The issue of retirement from athletic participation due to repetitive concussive injuries remains controversial. The complexity of providing recommendations to elite athletes is highlighted by the prospect that offering inappropriate advice may foreseeably lead to engagement in a medico-legal challenge. Currently no evidenced-based, scientifically validated guidelines for forming the basis of such a decision exist. The current paper discusses the complexities of this challenge in addition to presenting a case study of a professional athlete. A number of central issues to consider when discussing athlete retirement revolve around the player’s medical and concussion histories, the current clinical profile, the athlete’s long-term life goals, and understanding of the potential long-term risks. Ensuring that thorough investigations of all possible differential diagnosis, that may explain the presenting symptoms, are conducted is also essential. Discussion pertaining to recommendations for guiding the clinical approach to the retirement issue for athletes with a history of multiple concussions is presented.

## Introduction

Sports-related concussion and the potential long-term consequences for athletes with a history of participation in contact sports has received increasing focus following the publication of chronic traumatic encephalopathy case studies which have been based on autopsies of retired professional athletes (1, 2). Management of concussive injury in athletic populations have been largely guided, since 2002, by the development of guidelines (3, 4) that were subsequently modified via international consensus statements from the meeting of world experts in the field of sports-related concussion (5, 6). In addition to these guidelines, a number of governing bodies of collision sports have adopted mandatory, fixed duration, “sit-out” periods, as a means for addressing the potential risks of premature return-to-play of athletes (6). However, it is clear from the international consensus statements that an individualized and multifaceted management strategy should form the basis of best practice for concussion management. A related management concern, which has been largely bereft of evidence-based recommendations to guide the practitioner, is the issue of providing advice on athletic retirement following repeated concussive injury. Such decisions remain a complex and controversial area, not only due to an absence of evidence-based recommendations but also because of the possibility that providing inappropriate advice, at least at the professional level, may lead to engagement in a medico-legal challenge (7, 8). For example, even when valid evidence of cognitive impairment is found in an athlete’s neuropsychological assessment, a lawsuit seeking compensation to “payout” a lucrative contract that a player may have lost as a result of premature medical retirement advice may follow. However, it may also be likely that compensation will be sought for possible damages occurring after advice clearing the athlete to continue participating in contact sport. This concern is highlighted by the litigation brought before the courts in the USA in 2000 by a retired National Football League (NFL) running back, who successfully sued the physician employed by his former club for negligently failing to warn him about the dangers and risks of sustaining another and more severe concussion if he returned to play while suffering from post-concussion symptoms (9).

Unlike injuries to other regions of the body, where diagnosis, treatment, and the extent of recovery (prognosis) can be more readily objectively measured, athletic concussion is a unique injury, in that it involves a sacrosanct anatomical structure, the brain, that is vitally important to maintain a long-term quality of life (10, 11). In addition, the current controversies surrounding the level of scientific evidence pertaining to the potential long-term harms of exposure to concussive and sub-concussive injuries during an athletic career (12–14) promote a more conservative management approach than other (orthopedic) athletic injuries.

At the elite level the additional pressures generated through the “trial by media” where recommendations regarding the playing future of an athlete are compared to cases of athletes with reportedly poor outcomes following repeated concussions, often with little or no supporting medical evidence (7). The influence of the media has been highlighted recently with the frenzy surrounding the condition chronic traumatic encephalopathy (14), a disease process thought to be the result of exposure to repetitive head trauma in some individuals (1, 15). Currently, there is no scientific evidence that sustaining multiple concussions during a sporting career inevitably results in permanent damage (12, 13). In the absence of knowledge regarding the prevalence of CTE and a more comprehensive delineation of the risk factors involved, therefore, caution is necessary when making decisions regarding concussion management and statements about the likelihood that an athlete may progress to CTE. In view of these shortcomings, it appears imperative that acquiring objective evidence (i.e., neurological exam, neurocognitive data, neuroimaging, balance testing etc.), in addition to good clinical judgment and common sense remain the mainstay of concussion management (7).

The current medical literature is scarce in terms of published athlete case studies on the issue of retirement due to repeated concussive injuries. This paper presents a case study of a professional athlete with a history of multiple concussions during a 9 year elite level career, which highlights the complexities involved in providing retirement advice to athletes with a history of repetitive concussions.

## Background

The current case (referred to hereafter as “Case A”) is a 29-year-old right-handed, male, professional athlete, who presented for neuropsychological assessment at the conclusion of the athletic season with concerns regarding his history of multiple concussions and the potential long-term consequences they may have on his future.

Case A had made 122 first-grade appearances during a nine-season, first-grade career, primarily playing in positions that are known to have a greater incidence of concussive injury (16). He also had a history of playing at a junior level at the age of 7 years but no concussion record was available for his pre-professional years.

Case A completed 12 years of education, but indicated that he had always been a hyperactive individual and had experienced life-long difficulty paying attention for an extended period of time.

A recent brain MRI scan revealed a mild degree of cerebellar atrophy. It was also noted that the ventricles and CSF spaces were at the upper limit of normal for his age. There was no MR evidence of diffuse axonal injury or evidence of previous intracranial hemorrhage.

Case A expressed concern regarding his history of sports-related concussion and the potential impact that they may have on his future quality of life. While he denied experiencing any continued symptoms stemming from his most recent concussion, Case A indicated that he was sustaining concussions easier than he had previously. In addition, he reported that it was taking him longer to completely recover from them. He did not believe that increasing severity of the concussive injury was the reason for this apparent discrepancy.

In terms of his current cognitive function, Case A complained of a recent onset of forgetfulness that has caused him concern. For example, he indicated that he now loses track of what he is doing on tasks and makes simple errors in sending text messages on his phone. His fiancé also reported that she observed these lapses in his thinking.

Despite Case A’s history of orthopedic injuries/surgeries and osteoarthritis of the cervical and lumbar spine, he denied suffering pain symptoms. He denied problems with sleep or appetite and there was no reported history of psychiatric symptoms such as depressed or anxious mood, hallucinations, or delusions.

Case A reported that there was no known family history of neurodegenerative disease, learning disorder, or psychiatric illness.

### Documented concussion history

According to one of Case A’s club chief medical officer, he sustained numerous concussions each season during his time at the club, a number of which were described as substantial. Case A was considered particularly vulnerable to concussive injury by the team medical staff and also demonstrated a stiff neck and cervical spine problems.

Case A sustained four concussions during the most recent season. Based on the Case A’s team medical record, the first concussion in the most recent season resulted in a fractured zygoma, and a brief loss of consciousness. On interview, Case A denied suffering from residual post-concussion symptoms. According to Case A, the subsequent two concussions were mild and he did not suffer from any residual symptoms beyond the acute post-injury stage. The final concussion resulted in removal from play and he did not return. He suffered from a persisting headache the following day. On medical review at day 3 post-injury, Case A was observed to have difficulty concentrating on complex number and memory tasks and made numerous errors on balance/coordination testing. His Sport Concussion Assessment Tool 2 (SCAT-2) score was 66/100. This is considered to be quite a high score compared to normative data for the embedded tasks within the SCAT-2 (e.g., the total baseline Post-Concussion Symptoms Score (mean range = 3.5–6.5/22) (17), the firm surface Balance Error Scoring System (mean = 3.37/30) (18), the baseline Standardized Assessment of Concussion score (mean = 26.64/30) (19), and a Glasgow Coma Scale score; which by definition for a sports concussion should be a score of either 14 or 15/15). On day 5 post-injury, Case A suffered from an exacerbation of symptoms (dizziness) on exertion and he was not cleared to play the following week. He completed computerized neuropsychological screening which was normal compared to his individual preseason baseline performance. Case A was cleared to return-to-play but he was the victim of a high tackle in the first minute of the game. He was removed from play and on assessment in the dressing room during the acute stage post-injury was found to have no memory of the game, poor concentration and short-term memory for complex number recital, and poor balance.

### Self-reported concussion history

Case A reported that he had sustained over 10 previous concussions, the most recent of which occurred 1 month prior to the neuropsychological assessment conducted with him. Case A recalled that he had suffered one concussion eight seasons ago, two seven seasons ago, two five seasons ago, one or two four and three seasons ago, four or five last season and three during the most recent season. He indicated that at least 10 had resulted in amnesia, and approximately 8 had resulted in a brief loss of consciousness. He had never been hospitalized as a result of a concussion. Case A indicated that he had missed a total of 8 weeks from concussive injuries during his career, but that he had only experienced prolonged symptoms (greater than 1 week) on one occasion following a concussion but these resolved not long after (approximately 10 days).

### Results of previous computerized neuropsychological testing

Case A had undertaken six previous computerized neuropsychological screening tests (two baseline assessments and four post-injury assessments). There was evidence of changes between baseline and subsequent post-injury results but no evidence of a cumulative effect (see Table 1).

**Table 1:** **CogSport assessment results (*z*-scores)**.

	Ax 1: PI (866 days)	Ax 2: PI (851 days)	Ax 3: PI (816 days)	Ax 4: BL (636 days)	Ax 5: BL (94 days)	Ax 6: PI (87 days)
Detection	−0.76	−0.15	−0.28	−1.15	−0.55	−0.35
Identification	−0.10	0.08	0.08	0.37	−0.19	0.72
One card lrn	0.15	−1.44	−0.05	−0.42	−0.42	−0.24
One back	−0.09	−0.24	0.67	0.43	0.72	0.83
One back acc	2.24	1.09	2.24	0.64	1.09	0.64

Lrn, learning; Acc, accuracy; Ax, assessment; PI, post-injury; BL, baseline; days, number of days between testing and the current assessment.

### Self-reported questionnaire results

#### Current post-concussive symptoms

Case A completed a self-report inventory, pertaining to typical symptoms that may be experienced following head injury (Rivermead Post-Concussion Symptoms). On this symptom checklist, Case A endorsed mild impairment on a number of items (*n* = 10) including fatigue, irritability, feeling depressed or tearful, frustrated or impatient, forgetful, poor concentration, taking longer to think, blurred vision, light sensitivity, double vision, and restlessness. Total symptoms score was 10.

#### Psychological functioning

On a self-report questionnaire (the Depression Anxiety Stress Scale; DASS) pertaining to current psychological functioning, Case A endorsed a number of items. However his overall scores indicated current levels of depression, anxiety, and stress over the previous week were within the normal range compared to normative data.

#### Alcohol screen

There was a contrast between Case A’s in- and out-of-season level of alcohol consumption. He indicated that he very rarely consumed alcohol during the season but out-of-season he consumed much more, due in the main to the social functions he attends in the off-season. His in-season Alcohol Use Disorders Identification Test (AUDIT) score was 9; he reported consuming 10 or more standard drinks, monthly or less. His out-of-season AUDIT score was 20; he indicated that he consumed 10 or more standard drinks, two to three times per week.

### Neuropsychological assessment results

Case A’s assessment was conducted approximately 1 month after his most recent concussion. His performance on formal neuropsychological assessment revealed ability levels in accord with his estimated “low average” premorbid level of functioning across simple attention, processing speed, visuospatial construction, word knowledge, balance testing, and executive/adaptive functioning (verbal fluency, abstract reasoning, inhibitory control, and mental flexibility). In contrast, Case A’s performance on tests of learning and memory and fine motor skills were in the impaired range. These deficits were quite striking, as his performance on many of these task were in the bottom 2.2% of the population for his age and education level (see Table 2).

**Table 2:** **Neuropsychological assessment results**.

Test	Percentile
**WAIS-IV**	
Verbal Comprehension Index	27
Perceptual Reasoning Index	25
Working Memory Index	37
Processing Speed Index	23
Full Scale IQ	23
**WMS-IV**	
Auditory Memory Index	0.3
Visual Memory Index	3
Immediate Memory Index	37
Delayed Memory Index	58
Wechsler Test of Adult Reading	48
**REY–OSTERRIETH COMPLEX FIGURE**	
Copy	6–10
Immediate recall	<1
Delayed recall	<1
Recognition	1
**STROOP TEST COLOR-WORD TEST**	
Word Score	12
Color Score	4
Color-Word Score	35
Interference	68
**CONTROLLED ORAL WORD ASSOCIATION TEST**	
Letter fluency	19
Animal fluency	89
**TRAIL MAKING TEST**	
Trail making test A	43
Trail making test B	0.2
**GROOVED PEGBOARD**	
Grooved pegboard (dominant hand)	0.3
Grooved pegboard (non-dominant hand)	0.6
**BALANCE ERROR SCORING SYSTEM (BESS)**	
Balance error scoring system (firm surface)	42
Balance error scoring system (foam)	40

WAIS-IV, Wechsler Adult Intelligence Scale – Fourth Edition; WSM-IV, Wechsler Memory Scale – Fourth Edition.

## Discussion

### Clinical considerations and case formulation

Question 1: what is the relationship between Case A’s observed memory impairment and his history of multiple sports-related concussions?

While memory difficultly was certainly demonstrated on current testing, along with impairment of divided attention (i.e., Trails B); other aspects of cognition typically affected by a history of concussive injuries [such as attentional capacity (20), processing speed (21), and executive functions like problem solving, decision-making, inhibitory control, mental flexibility, planning, and organization] were are all within normal limits.

Question 2: what is the relationship between the neuroimaging evidence of atrophy and Case A’s history of the repeated concussive and sub-concussive blows?

The reported neuroimaging atrophy is concerning and does raise suspicion that it may be sequelae of a considerable concussive history, as no further medical condition has been found to explain the cause.

Question 3: what clinical recommendations are appropriate if relationships identified in questions one and two are assumed to be causative?

If either, or both, the cognitive deficits and/or the neuroimaging pathology are considered to be a consequence of Case A’s concussion history, then consideration to reduce the exposure (i.e., potential retirement) is recommended. However, in view of the current anecdotal research evidence in this area (1, 2), it is purely speculative to predict any delayed onset of long-term adverse effects or to comment on the potential quality of life Case A may have in the future.

Further, the evidence that Case A had sustained more frequent concussive injuries as a result of less forceful impacts, and requiring longer recovery periods from these seemingly innocuous blows, raised concern regarding his vulnerability and the possible longer term effects of further exposure. These set of circumstances have been reported to be indicators for retirement (22, 23).

### Who are the stakeholders involved in the retirement decision-making process of professional athletes?

In the case of Case A, the player consulted his employer (i.e., his club), club medical staff, his player agent and his family, who all made considered contributions in light of the neuropsychological and medical evidence presented to them. While this level of consultation did not result in confrontation in the instance of Case A, the strategy of consulting stakeholders who possess an obvious conflict of interest (and other ethical concerns; such as those discussed below) may not always result in a positive outcome for the athlete.

In terms of the clinical interpretation of Case A’s profile and the feedback and recommendations presented to him, a conservative approach was adopted which highlighted the concern regarding his apparent greater susceptibility to concussive injury, increased severity of symptoms, and the longer recovery from these symptoms with each successive injury. These concerns, together with the memory deficits on neuropsychological assessment and neuroimaging evidence of atrophy on a background of uncertainty regarding the possible long-term consequences of concussive injury formed the foundation for the recommendation to retire from contact sports. Ultimately this occurred, Case A made the decision himself to retire from professional sport and pursue a career in the fitness industry.

While international consensus recommendations are available for the management of concussion (6), often these do not apply to the retirement decision and guidelines or recommendations pertaining specifically to the decision of retirement from athletic competition due to repetitive concussion are relatively absent. In 2003, Cantu (23) discussed “absolute contraindications” and “relative contraindications” for retirement, based largely on the athlete’s response to concussion (i.e., duration and severity of symptoms, and ease of which they were concussed) rather than the number of concussions they had experienced during their career. In contrast, Sedney et al. (24) focused on the important implication of a history of concussive and sub-concussive blows (diagnosed and undiagnosed concussion, respectively) over an extended period of time. These proposed guidelines were broken down into season ending recommendation and career ending recommendations but were far more prescriptive, less individualized and therefore less flexible. For example, an athlete with three or more “major” concussion would be encouraged to terminate their career.

There are a number of important aspects that require consideration when engaging in this clinical decision-making process. The challenges creating uncertainty in the present case involved;

a) complete recovery was reported from all previous post-concussive symptoms;
b) normal performance across the computer-based cognitive testing format;
c) non-specific neuroimaging abnormality.

These challenges occur on the background of developing (yet to be delineated and established) knowledge regarding the potential for long-term consequences (e.g., chronic traumatic encephalopathy) of repetitive concussion.

### Clinical guidance for considering retirement

Amidst the uncertainty surrounding retirement decision-making, and in the absence of guidance from the medical literature, together with the ongoing importance placed on good clinical judgment and common sense, the following approach was applied in this case and may provide guidance for other similar cases.

#### A. Concussion history

Obtain a thorough understanding of the documented and self-reported concussion history, with particular emphasis on:

- number of previous concussions;
- cause of the concussion;
- concussion symptom(s) severity and duration, with the view to determining whether there is evidence of greater susceptibility and consequent of successive injuries;
- return-to-play following each incident (i.e., recovery);
- objective data obtained (e.g., computerized neuropsychological test results, balancing test results, neuroimaging).

#### B. Current clinical profile

Consideration of the current clinical profile including:

- determining whether recovery from the most recent neurological event has occurred, or whether the current clinical profile represents post-concussive sequelae, and therefore recovery may be anticipated;
- documenting current post-concussive symptoms;
- obtaining neuropsychological assessment data;
- collecting balance testing data;
- neuroimaging investigation data;
- mood and/or psychological state;
- psychiatric symptoms and/or history.

#### General common sense issues

- collect a thorough medical history, including but not limited to
  - developmental history (learning and education, etc.);
  - neurological conditions or other medical conditions (including any other brain injuries);
  - alcohol and other drug use;
  - psychiatric and psychological history;
  - possible genetic contributors (i.e., a family history of dementia or learning disorder);
  - assessment for possible chronic pituitary dysfunction, thyroid dysfunction, or adult growth hormone deficiency (GHD); for a proposed screening strategy, see Tanriverdi et al. (25).
- ensure that the presenting symptoms are not readily explained by other neurological or medical conditions or disorders, and therefore may potentially be treatable.
- continued exposure to brain trauma is unlikely to improve any current post-concussive symptoms.

#### D. The patient/athlete

The athlete brings with them multiple non-medical related aspects which may be of equal value and require consideration, including:

- sporting (and non-sporting) career opportunities and goals;
- a level of comprehension regarding the potential risk of participation, in view of their own concussive history;
- other (non-neurological) injury history and recovery, together with a level of resilience and determination to return-to-play;
- personality and/or behavioral style/traits (e.g., impulsive or a risk taker).
- Financial management and future occupational planning – it may be the case that the athlete may never earn as much income as they have playing professional sports.

While many cases of athletic retirement have been reported in the popular press/media, very few are described in the medical literature. Many of these cases refer to post-concussion syndrome (prolonged recovery from an athlete’s most recent concussion). The current reported case is unique in that Case A subjectively reported a full recovery from his most recent concussion, and that his ongoing concerns (although he felt related to his history of multiple concussions), were not simply residual post-concussion sequelae from the recent episode.

A number of aspects in this case were important for a favorable outcome, which may not always be present in every instance of athletic retirement decision-making. Firstly, Case A demonstrated appropriate insight into his deficits and was open to receiving guidance from medical staff. Secondly, Case A was future- and not present-focused, as such he was cognizant of the potential that his history of repetitive concussion may have detrimental long-term consequences. This may have been a reflection of his age and level of experience at the elite level. That is, he was in the latter stages of his career where post-athletic career options were likely already being considered by him. Younger, or early career players, may be less likely to be future focused. Young men are also more likely to consider themselves “bullet proof” and may be insightless to the potential risks involved, which was certainly not the case with Case A. Finally, notwithstanding the intact computerized neuropsychological testing, Case A possessed obvious cognitive deficits on more formalized neuropsychological assessment, which not only served to provide insight to Case A, but also formed the basis for concern. In other cases were more subtle cognitive deficits are present, the formulation of recommendations is far more challenging.

## Concluding Remarks

The decision of retirement from contact sport should always be made independently and without coercion, but with appropriate education and recommendations for the athlete to make an informed decision. Whether the player seeks and adheres to medical advice is entirely the prerogative of the athlete. Where investigation into possible problems is conducted, the neuropsychological assessment and post-concussive symptom severity and duration data, can provide an important source of information to assist with this decision as it may allow for a comparison between premorbid or pre-injury baseline data and current functioning. It is essential that a thorough investigation of all possible causes of the presenting symptoms is conducted, for example, neuroendocrine examination for pituitary dysfunction or adult GHD. These entities are not only known to be characterized by similar sequelae as post-concussion symptoms and therefore may be missed and untreated, but they are also known to be prevalent after repetitive concussions (26). Of further clinical importance, considerable consequences may result from a missed diagnosis (27). In Case A, his neuropsychological assessment performance raised concern regarding continued exposure to concussive injury and in view of the striking memory deficits provided sufficient evidence for Case A to seriously consider retirement from contact sport. Computerized measures of cognitive ability, as reported on for Case A, have been suggested as a more sensitive method for detecting processing speed and attention problems that may be outside an athlete’s awareness or considered as unimportant by the player (28, 29). However, the irregular use of computerized neuropsychological screening may have limited their usefulness in Case A. Mandatory fixed duration, sit-out periods, or retirement recommendations based upon concussion counts (i.e., a “three strike rule”) lack scientific merit, instead assessment, management, and decision-making based on individual circumstances and based on the current consensus guidelines (6) is recommended.

In the case of Case A, his neuropsychological assessment performance raised concern regarding continued exposure to concussive injury and in view of the striking memory deficits provided sufficient evidence for Case A to seriously consider retirement from contact sport.

## Conflict of Interest Statement

The author declares that the research was conducted in the absence of any commercial or financial relationships that could be construed as a potential conflict of interest.
